# Clozapine: Why Is It So Uniquely Effective in the Treatment of a Range of Neuropsychiatric Disorders?

**DOI:** 10.3390/biom11071030

**Published:** 2021-07-15

**Authors:** Dara Gammon, Catherine Cheng, Anna Volkovinskaia, Glen B. Baker, Serdar M. Dursun

**Affiliations:** 1 Saba University School of Medicine, Saba, The Netherlands; daragammon@gmail.com (D.G.); annavolkovinskaia@gmail.com (A.V.); 2 Neurochemical Research Unit and Bebensee Schizophrenia Research Unit, Department of Psychiatry, University of Alberta, Edmonton, AB T6G 2B7, Canada; ccheng5@ualberta.ca (C.C.); glen.baker@ualberta.ca (G.B.B.); 3 Department of Psychiatry, University of Toronto, Toronto, ON M5T 1R8, Canada; 4 Neuroscience and Mental Health Institute, University of Alberta, Edmonton, AB T6G 2E1, Canada

**Keywords:** clozapine, antipsychotics, electroconvulsive therapy, schizophrenia, suicide, bipolar disorder, major depressive disorder, Parkinson’s disease

## Abstract

Clozapine is superior to other antipsychotics as a therapy for treatment-resistant schizophrenia and schizoaffective disorder with increased risk of suicidal behavior. This drug has also been used in the off-label treatment of bipolar disorder, major depressive disorder (MDD), and Parkinson’s disease (PD). Although usually reserved for severe and treatment-refractory cases, it is interesting that electroconvulsive therapy (ECT) has also been used in the treatment of these psychiatric disorders, suggesting some common or related mechanisms. A literature review on the applications of clozapine and electroconvulsive therapy (ECT) to the disorders mentioned above was undertaken, and this narrative review was prepared. Although both treatments have multiple actions, evidence to date suggests that the ability to elicit epileptiform activity and alter EEG activity, to increase neuroplasticity and elevate brain levels of neurotrophic factors, to affect imbalances in the relationship between glutamate and γ-aminobutyric acid (GABA), and to reduce inflammation through effects on neuron–glia interactions are common underlying mechanisms of these two treatments. This evidence may explain why clozapine is effective in a range of neuropsychiatric disorders. Future increased investigations into epigenetic and connectomic changes produced by clozapine and ECT should provide valuable information about these two treatments and the disorders they are used to treat.

## 1. Introduction

### 1.1. Clozapine

Clozapine was first developed over 50 years ago as a potential pharmacological therapy for the treatment of schizophrenia [1,2,3]. Clozapine was noted to differ from other antipsychotics available at that time because it produced fewer extrapyramidal symptoms (EPS); it was also reported to have marked analgesic potential [1]. It was coined as the first atypical antipsychotic but was not used extensively and was withdrawn from the market after reports of agranulocytosis in the 1970s [1]. Clozapine was later reintroduced after two studies published in the late 1980s showed its efficacy in a significant number of treatment-resistant patients with schizophrenia [4,5], and in 1990 it became an FDA-approved antipsychotic in the United States. Although highly effective, clozapine remains an underprescribed medication, limited by the requirement for frequent laboratory monitoring, a regimented titration schedule, and a severe side effect profile, which includes the risk of agranulocytosis, myocarditis, seizures, constipation, arrhythmia, syncope, hypersalivation, pneumonia, obsessive–compulsive symptoms [6,7,8], and metabolic syndrome consisting of the dysregulation of glucose, insulin, plasma lipids, and body fat [9].

**Figure 1:**
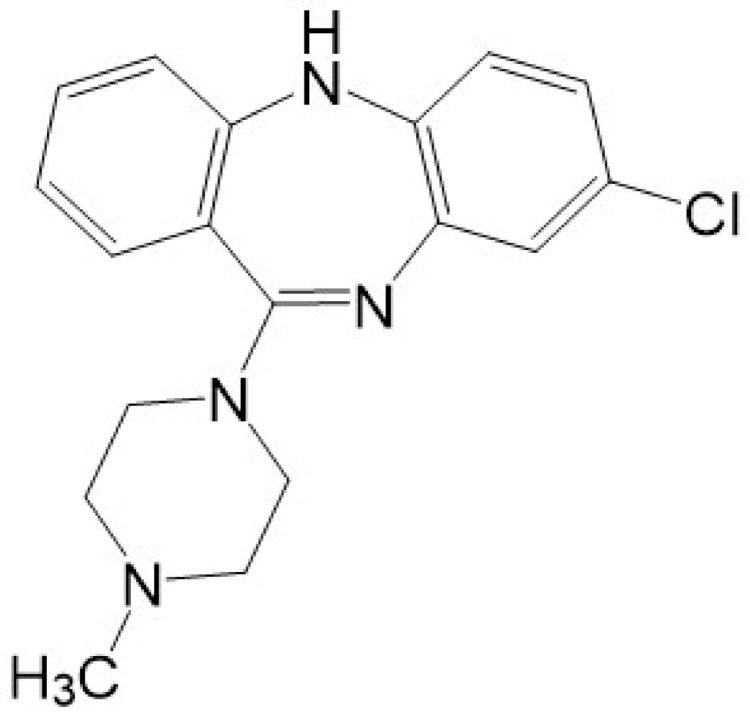
Chemical structure of clozapine.

Clozapine is a tricyclic dibenzodiazepine (Figure 1). The exact mechanism of its antipsychotic action is still unknown, but it is well documented that clozapine affects many neuroreceptors in the brain [10,11,12]. It is an antagonist at the dopamine receptors (affects D2 receptors weakly and loosely, with less than 60% occupancy, and has a high affinity for D4 receptors) and also binds with a high affinity to multiple serotonin receptors (5-HT1A, 5-HT2A-C, 5-HT6, 5-HT7), alpha 1 (α1) and alpha 2 (α2) adrenergic receptors, histamine (H1) receptors, and M1–M5 muscarinic receptors [2,10,11]. Clozapine’s relatively rapid dissociation from D2 receptors [13] and its antagonistic activity at the 5-HT2A receptors [11] have been put forward as mechanisms responsible for its effectiveness as an antipsychotic, and its actions at multiple receptors account for many of its adverse effects [14].

Clozapine has a major metabolite, N-desmethylclozapine (NMDC, norclozapine), which has activity comparable to clozapine at the D2 and 5-HT2A receptors as well as at several other receptors [10]; both the metabolite and parent drug can activate N-methyl-D-aspartate (NMDA) glutamate receptors [10]. Clozapine is acted on by several CYP enzymes, with CYP3A4 and CYP1A2 primarily responsible for N-demethylation to NMDC and CYP1A2 considered to be the principal catalyst for the formation of clozapine-N-oxide (CNO). Smoking, which can induce CYP1A2, can result in reduced serum concentrations of clozapine, whereas administration of the antidepressant fluvoxamine, a strong inhibitor of CYP1A2, increases serum levels of clozapine. Although it has been proposed that NMDC is an active metabolite contributing to the therapeutic effect of clozapine, it may also contribute to reduced neutrophil counts in patients with schizophrenia taking clozapine [15]. CNO has been considered an inactive metabolite, but it has been reported that both NMDC and CNO are more neuroprotective than clozapine in neuron–glia cultures treated with the neurotoxic agents lipopolysaccharide and 1-methyl-4-phenyl-1,2,3,6-tetrahydropyridine (MPTP) and that the neuroprotective effect is the result of the inhibition of microglia-mediated neuroinflammation [16].

According to the Food and Drug Administration (FDA) of the USA, clozapine has two approved indications for use. The first is for patients with treatment-resistant schizophrenia previously taking adequate doses of other antipsychotics (a trial of two or more, with at least one being an atypical (second generation) antipsychotic). The second is to reduce further risk of self-harm in patients with recurring suicidal behavior diagnosed with either schizophrenia or schizoaffective disorder [17]. Clozapine has also been used off-label in the treatment of Parkinson’s disease (PD), bipolar disorder, and schizophrenia with comorbid depression [18]. There is also preliminary evidence that it may be an effective alternative therapy in unipolar major depressive disorder (MDD) [19].

**Figure 2:**
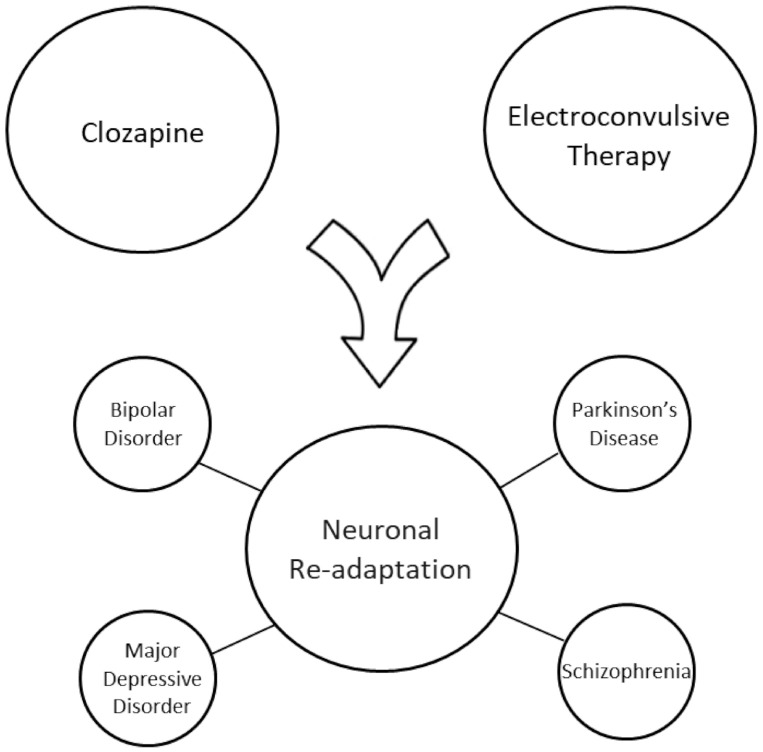
Neuropsychiatric disorders in which both clozapine treatment and ECT have been reported to be effective.

The goal of this review was to assess the literature in an effort to identify potentially unifying mechanisms of action between clozapine and electroconvulsive therapy in their ability to effectively treat a range of psychiatric and neurologic illnesses, including schizophrenia, MDD, bipolar disorder, and PD. We hypothesized that common or related mechanisms of action exist between these two treatment modalities that may be related to their ability to treat a range of neuropsychiatric disorders (Figure 2).

### 1.2. Electroconvulsive Therapy (ECT)

Electroconvulsive therapy (ECT) is a nonpharmacological neurostimulation method that has been found to be effective in treating ultra-resistant and severe forms of several mental illnesses such as depression and bipolar disorder [20,21,22]. ECT is often offered as treatment of last resort when other therapies have failed and/or when rapid reduction of often severe symptoms is required, e.g., suicidal ideation and catatonia [21]. ECT is a procedure that applies electrical stimulation to induce controlled, brief generalized seizures while patients are under anesthesia and administered a muscle relaxant [22]. As is the case with clozapine, the mechanism of action of ECT is not completely understood and appears to be multifaceted [20]. In animal models, an equivalent procedure known as electroconvulsive shock (ECS) has been shown to increase the volume of specific brain areas, an effect associated with improved behavior and increased neuroplasticity [22]. Other theories on the mechanism of action of ECT/ECS include: the induction of neuroendocrine changes such as the rapid release of adrenocorticotropic hormone, prolactin, and cortisol into the blood [23] and effects on neuroplasticity, including axonal sprouting and synaptic reorganization by affecting the balance between mGluR1/5 and AMPA glutamate receptors, resulting in the subsequent release of brain-derived neurotrophic factor (BDNF) by AMPA receptors and, ultimately, promotion of neurogenesis [20,24].

### 1.3. Clozapine, Seizures, and Neurogenesis

To look further into identifying possible mechanisms of action of clozapine in the treatment of various psychiatric illnesses, we must discuss clozapine’s ability to alter signaling within the brain. As part of its side effect profile, clozapine is known to lower the seizure threshold and may induce electroencephalogram (EEG) changes, myoclonus, and generalized tonic-clonic seizures [25,26,27,28]. In a review of 12 papers, Varma et al. [26] noted that many patients taking clozapine were reported to have EEG changes, with reported incidences ranging from 25 to 100%; the most common EEG change was generalized slowing involving delta and theta waves. In a systematic review of EEG changes in patients on antipsychotic therapy, Jackson and Seneviratne [28] identified eight studies (pooled n = 229) involving the use of clozapine and found an association with both EEG slowing and epileptiform discharges, and reported that clozapine therapy increased the odds of the occurrence of epileptiform discharges on EEG by 6-fold. In studies by Stevens et al. [25,29], myoclonic jerks/seizure activity in rats after repeated low doses of clozapine increased central nervous system excitability or “kindling” in the ventral tegmental area, an area crucial for regulating dopamine pathways in the brain, and in the anterior thalamic nuclei. The excitation or “kindling” subsequently resulted in myoclonus [29]. The expression of the immediate early gene c-fos was also increased in these two brain areas in the clozapine-treated animals.

Clozapine’s annual seizure risk in humans reported by the manufacturer is 3.5% versus 1% with conventional (typical) antipsychotics [26]. For a summary of the incidence of seizures and EEG abnormalities reported in the literature for clozapine, see [26]. Denney and Stevens, in a study on the effects of clozapine on myoclonic jerks in partially restrained rats, concluded that clozapine might exert its therapeutic effect by causing an increase in excitability in critical subcortical areas of the brain, which produces the antipsychotic effect. These authors also noted that there is clinical evidence indicating that clozapine-induced paroxysmal epileptiform EEG changes are associated with clinical improvement even in the absence of convulsions in the patient [30].

As mentioned previously, clozapine acts on many types of neuroreceptors in the brain and is rather unique in that it has a higher affinity for receptors other than the D2 receptor, i.e., D1, D3, and D4 receptors and a variety of serotonin, muscarinic, histamine, and noradrenergic receptors [10,12,14]. However, it should be considered that clozapine’s efficacy may not only be due to its ability to block numerous neuroreceptors but also to the EEG changes it induces, an action similar to manually stimulating the brain using an electrical current, as in the case of ECT [28,29].

Clozapine also activates extracellular signal-regulated kinases (ERK1/2), which are involved in the regulation of transcription and in improving synaptic plasticity, connectivity, and neurogenesis [31]. It has also been reported to enhance the expression of neurotrophic factors such as brain-derived neurotrophic factor (BDNF), an effect it shares with ECT, which may make it superior to other antipsychotic medications [32]. Clozapine is believed to induce neurogenesis through improved neuronal cell survival, increased expression of BDNF, and restoration of neuronal architecture in the dentate gyrus, with dendritic length equaling that of controls after clozapine administration in animal models of schizophrenia [33,34].

We will now examine the various disease states treated by both clozapine and ECT, present current findings, and review proposed mechanisms of action behind their therapeutic use.

### 1.4. Search Strategy

This narrative review identified and investigated studies regarding the application of clozapine and ECT in the treatment of schizophrenia spectrum disorders, bipolar disorder, MDD, and PD and discusses potential common or related mechanisms behind their effectiveness. A comprehensive literature search was conducted, and relevant articles up until March 2021 were screened. PubMed, ClinicalTrials.gov, and PsychInfo databases were searched using the following combination of terms: “clozapine, electroconvulsive therapy, mechanism of action, seizures, schizophrenia, bipolar disorder, major depressive disorder, and Parkinson’s disease.” Further articles were identified for inclusion by examining reference lists in the papers screened.

## 2. Effects of Clozapine and ECT on Neuropsychiatric Disorders

### 2.1. Clozapine and Schizophrenia Spectrum Disorders

Clozapine is the gold standard for treatment-resistant schizophrenia and schizoaffective disorder [14]. Although a dopamine receptor blockade may be a necessary component in the treatment of schizophrenia, it is not sufficient in one-third of patients who are termed treatment-resistant and have an indication for a trial of clozapine [34,35]. Of the currently available antipsychotics, clozapine has been found to have the lowest affinity for the D2 receptor, but it is also known to block many additional receptors that may contribute to its antipsychotic properties, such as the 5HT2A receptors and D4 receptors [36]. In a meta-analysis of clinical and demographic features as predictors of clozapine response in patients with schizophrenia spectrum disorders, Okhuijsen-Pfeifer et al. [37] identified 34 articles and found that younger age, fewer negative symptoms, and paranoid schizophrenia subtype were associated with a better clozapine response. Although clozapine is known to antagonize various dopamine receptors and the 5-HT2A receptor and act on several other neurotransmitter receptors, which contribute to its side effect profile [2,10,11], its other mechanisms of action that set it apart from other atypical antipsychotics are not well understood. Nair et al. [38] hypothesized that, similar to the GABA agonist and muscle relaxant baclofen, clozapine may bind directly to the GABA_B_ receptor, a receptor that may play a role in the pathogenesis of schizophrenia since decreased GABA_B_ receptor expression has been reported in animal models of schizophrenia and in several brain areas in postmortem tissue from patients with schizophrenia. In a prospective-longitudinal transcranial magnetic stimulation (TMS) study into cortical inhibition in treatment-resistant schizophrenia (n = 16), clozapine was also found to be associated with an increase in GABA_B_ receptor-mediated inhibitory transmission [39]. A study by Marx et al. [40] found that clozapine markedly elevated levels of the neuroactive steroid pregnenolone in the rat hippocampus, cerebral cortex, and serum (13-, 26- and 34-fold, respectively), suggesting that the induction of pregnenolone, which improves learning and memory in rodents, may contribute to the clinical actions of clozapine. Pregnenolone is also a precursor for several other neuroactive steroids that act as allosteric modulators at GABA_A_ receptors and/or NMDA glutamate receptors. Clozapine has also been reported to elevate levels of the strong positive GABA_A_ receptor allosteric modulator allopregnanolone in the rat cerebral cortex [41]. Clozapine also affects the glutamate/GABA imbalance that is present in many neuropsychiatric disorders via actions on a variety of glutamate receptors [42,43,44,45].

Reversal of dopamine receptor supersensitivity is another proposed mechanism by which clozapine may exert its effect in treatment-resistant schizophrenia [46]. Animal studies also suggest that clozapine may reduce comorbid substance use in patients with schizophrenia, possibly because of its combined properties as a weak D2 receptor antagonist, potent α-2 receptor antagonist, and norepinephrine reuptake inhibitor [6].

### 2.2. ECT Augmentation in Schizophrenia Spectrum Disorders

ECT has been used as a treatment since 1939; historically, it was used on its own for the management of psychotic disorders until the advent of chlorpromazine and other antipsychotic drugs. Currently, the most frequent use of ECT on its own for psychotic disorders is in developing countries. There is a general consensus among various professional associations worldwide that ECT can be useful in schizophrenia for catatonia, psychotic exacerbation, suicidal behavior, and poor response to antipsychotics [47]. This last-mentioned use will be discussed in this section.

There is growing evidence to support the use of ECT as an augmentation strategy for individuals with little improvement on clozapine, termed ultra-resistant schizophrenia (URS) [48,49,50]. In a meta-analysis of 18 randomized controlled trials, Wang et al. [51] concluded that ECT augmentation of clozapine in clozapine-resistant schizophrenia is highly effective and relatively safe. Grover et al. [49] investigated the effectiveness of ECT in clozapine-resistant schizophrenia and non-clozapine-resistant schizophrenia and reported that ECT was an effective augmentation strategy for both groups. In a retrospective case series study, Kim et al. [52] reported that ECT induced remission in 71.4% of patients with clozapine-resistant schizophrenia; these patients had at least a 20% reduction in total Positive and Negative Syndrome Scale (PANSS) scores, but ECT did not reduce the negative subscale score on the PANSS in any patient. In a clinical case series study of nine patients with URS using ECT as a potentiation strategy with clozapine, ECT was found on average to result in a reduction of 29.8% in the Brief Psychiatric Rating Scale (BPRS) score [48]. Based on these findings, Rotharmel et al. [48] recommend that ECT treatment in this population should ideally be twice a week and consist of 16–20 sessions (with maintenance sessions if aggressive behaviors are present). In a systematic review of the safety and efficacy of combining brain stimulation techniques with clozapine, Arumugham et al. [53] found that ECT is an effective intervention in clozapine-refractory schizophrenia but stated that there was insufficient evidence at that time to support the safety of the combination therapy. A Cochrane review on the use of ECT in treatment-resistant schizophrenia found moderate-quality evidence that ECT has a positive effect on medium-term clinical response compared with standard care; however, the authors concluded that more quality evidence is required to determine if augmentation with ECT is superior and if the use of ECT alone should be supported or refuted [54]. Lally et al. [50] conducted a retrospective review of electronic health records and reported that the results supported the emerging evidence for the effectiveness of the combination of clozapine and ECT in clozapine-resistant schizophrenia. Moulier et al. have published a study protocol in which they propose to study the following parameters of combined clozapine-ECT treatment in URS closely: optimal number and frequency of ECT sessions and the relevance of maintenance ECT [55].

An international initiative was undertaken by the Treatment Response and Resistance in Psychosis (TRRIP) working group to develop consensus recommendations for the management of treatment-resistant schizophrenia with inadequate clozapine response [56]. The group achieved a consensus in recommending combination with a second antipsychotic (amisulpride or oral aripiprazole) and augmentation with ECT for clozapine-refractory positive symptoms; augmentation with antidepressants or mood stabilizers and ECT met the consensus criteria for clozapine-refractory suicidality.

Despite growing evidence of synergistic effects with clozapine, ECT augmentation remains a promising yet underutilized therapy for treatment-resistant schizophrenia [49,57].

### 2.3. Clozapine and Bipolar Disorder

Currently, the majority of the literature describing the off-label use of clozapine is in the treatment of bipolar disorder [58,59,60,61]. Historically, acute mania has been treated with clozapine since the 1990s [61]. The 2018 Canadian Network for Mood and Anxiety Treatments (CANMAT) and the International Society for Bipolar Disorders (ISBD) guidelines for the management of bipolar disorders currently recommend clozapine as a third-line agent for the treatment of mania and as a potential adjuvant in maintenance treatments [62]. In a review of off-label pharmacotherapies in treatment-resistant bipolar disorders, Poon et al. [60] noted that clozapine does show some evidence of efficacy in the treatment of refractory mania, with or without psychotic symptoms, when given alone or as an adjunctive medication. Similarly, a systematic review of 15 studies (n = 1044) by Li et al. [58] found that, although limited, current evidence supports the use of clozapine as an effective and relatively safe medication in treatment-resistant bipolar disorder. Kapczinski et al. [63] highlighted a similar clinical course, neural substrates, and neurobiology between schizophrenia and type I bipolar disorder, the former for which clozapine is a gold standard treatment in treatment-resistant cases. In summarizing the results of recent systematic reviews and trials, while acknowledging the limited number of randomized controlled trials, Kapczinski et al. [63] found that clozapine improved mood and psychotic symptoms, reduced the number and duration of hospitalizations, reduced the number of psychotropic medications taken, reduced self-harm, suicidal ideation, and aggressive behavior, and improved social functioning in treatment-resistant bipolar disorder. In a case series of nine patients with chronic and treatment-resistant bipolar I disorder, clozapine therapy was reported to be successful in improving symptoms of mania and mood lability, with a low mean clozapine dose at the study endpoint of 156.3 ± 77.6 mg/day and duration of treatment of 12 months [64]. In a systematic review of the use of clozapine in bipolar disorder, Delgado et al. [65] identified nine studies of patients treated with clozapine (n = 100) suitable for meta-analysis; they found that clozapine appeared to be faster at improving symptoms of mania than other antipsychotics and had an overall efficacy similar to other antipsychotics in the treatment of manic episodes. A systematic review of clozapine effectiveness in bipolar disorders and primary psychotic disorders in older adults identified seven studies (n = 128) and found that clozapine may have positive effects, but acknowledged the limitations of this finding, including modest group effects, low-level evidence, and methodological limitations [66]. There is also some evidence to support the adjunct use of clozapine in treatment-resistant bipolar patients with severe suicidal ideation. Low-dose clozapine therapy was reported to be effective in improving symptoms in a case series of three treatment-resistant bipolar patients with severe suicidal ideation and was observed to have notable anti-suicidal effects [67]. Despite its effectiveness, the mechanisms by which clozapine exerts its effects in bipolar disorder are currently not fully understood. In a retrospective study of patients who had been treated with clozapine, Pillay et al. [68] found that patients with major depressive episodes (bipolar, schizoaffective, unipolar) and abnormal EEG traces were likely to have a favorable response to clozapine. In a review by Maletic and Raison on the integrated neurobiology of bipolar disorder, it was proposed that impairment in neuroplasticity may be part of the pathogenesis of this disorder, which is supported by changes observed in bipolar patients’ accelerated loss of volume in brain areas important in the regulation of mood and cognitive function, and changes at the cellular level indicating the dysregulation of glial–neuronal interactions, with overactivity of microglia and increased inflammation [69]. Wilkowska and Cubala [32] described clozapine as a potentially transformative treatment in bipolar disorder, citing its unique pharmacology as well as epigenetic and neuroplastic effects as distinguishing factors.

### 2.4. ECT and Bipolar Disorder

Despite variances across the globe with regard to guidelines for the treatment of bipolar disorder, ECT plays a role in the majority of guidelines as a second-line option for treatment-resistant or refractory mania and as a first-line modality in severe bipolar I disorder, psychotic depression, and elevated suicidal risk [70]. The American Psychiatric Association (APA) practice guidelines recommend the use of clozapine or ECT as second-line therapeutic options if patients have treatment-resistant bipolar disorder. ECT has been used in the treatment of mixed bipolar disorder (simultaneous occurrence of both mania and depressive symptoms), treatment-resistant mixed states [71], and treatment-resistant bipolar depression [72]. In a study of predictors of ECT response in individuals with bipolar depression (n = 670), 72% of patients were responders, and those with psychomotor disturbances, mood-congruent delusions, and severe mixed episodes were highly responsive to ECT treatment [73]. In a naturalistic study following bipolar patients after successful ECT treatment, with a mean follow-up duration of 57 weeks, Medda et al. [74] found that 93% of patients (n = 70) maintained at least a partial response for more than 90% of the follow-up period and 73% of patients fulfilled criteria for full remission. Further studies are required to evaluate the long-term efficacy of ECT in bipolar disorder.

In a review of ECT use in severe bipolar mixed states, Perugi et al. [75] found that ECT was effective in patients nonresponsive to pharmacotherapy; however, offering ECT was often delayed and considered a treatment of last resort, which may decrease chances of recovery in patients who may have responded if ECT was offered in a timely manner. Morcos et al. [76] conducted a retrospective study of older adults with bipolar depression who received ECT (n = 34), and ECT was found to be well tolerated and effective in treating depression. The authors recommended that regular consideration for ECT should be given for older adults with bipolar depression who have not responded to pharmacotherapy. A systematic review by Calaway et al. [77] also validated ECT’s safety in pregnant patients with various psychiatric illnesses, including bipolar disorder. In a comprehensive review of ECT use in mania by Elias et al. [78], several additional mechanisms of action are discussed, with some parallels with clozapine. One proposed mechanism by which ECT exerts its effects in the treatment of mania may be through increasing the seizure threshold, which occurs with an increasing number of treatments; this increased seizure threshold is associated with the degree of improvement in manic symptoms, and relapse may occur upon return to the baseline seizure threshold. Additional proposed mechanisms include inhibitory effects on membrane excitability through a reduction in regional cerebral blood flow and metabolic rate for glucose in the prefrontal cortical regions and increased slow-wave activity on EEG post-ECT. Neurochemically, there may be an increase in GABA levels, which has previously been found to be decreased in bipolar disorder. This GABA-elevating action is a property shared with other pharmacotherapies, including the mood stabilizers lithium and valproate [78]. Currently, the exact mechanisms by which ECT exerts its effects in bipolar disorder remain largely unknown, although additional effects may include: counteracting oxidative stress, glutamatergic dysfunction, and dysfunctional serotonergic transmission; increasing levels of neurotrophic factors; and activating the mesocorticolimbic dopamine system [79,80].

### 2.5. Clozapine and Major Depressive Disorder (MDD)

The average adult has been proposed to have a 5–26% likelihood of having a major depressive episode in their lifetime [81]. Depression worsens morbidity, increases the likelihood of mortality, and decreases quality of life in general and is a prevalent psychiatric illness that can present independently or exist comorbidly with other psychiatric disorders [82]. Clozapine is not traditionally used as a primary treatment for MDD, but a large study by Tiihonen et al. [19] on unipolar MDD found that clozapine significantly reduced the risk of hospital readmission in patients with MDD; in fact, lithium and clozapine were substantially more effective in this regard than antidepressants and antipsychotics such as aripiprazole and quetiapine. Clozapine has been studied in chronic schizophrenia patients with depression and appears superior to quetiapine in treating symptoms of depression in these patients [82].

Clozapine has also been shown in a rat model of depression to improve behavioral despair and anhedonia, re-establish stress-induced neuronal structure and gene expression impairments in the hippocampus and prefrontal cortex, and upregulate neurogenesis and neuronal survival, a property it shares with traditional antidepressant medications [83]. Antidepressant treatments are also believed to exert, at least in part, their effects through increasing levels of neurotrophins, such as BDNF, in the brain; clozapine has been found to increase BDNF levels by 8–10% in the frontal cortex, although this is a smaller increase than that produced by first-line antidepressant medications (10–30%) and repeated ECT (40–100%) [84].

### 2.6. ECT and Major Depressive Disorder (MDD)

About one-third of patients with depression are non-responders to pharmacological treatment with currently available antidepressant drugs [85]. ECT is considered the most effective treatment for MDD [86] and has been shown to be safe and well tolerated in various patient populations, including geriatric patients with severe depression [87]. In a study comparing efficacy, safety, and tolerability of formula-based right unilateral ECT (RUL) versus bilateral ECT (BT) in the treatment of unipolar major depression, Dominiak et al. [88] indicated that ECT remains the most effective treatment of depression and that RUL and BT are equivalent in their antidepressant efficacy (n = 91), with RUL being safer and having greater tolerability. Farzan et al. [89] reviewed and discussed the mechanism behind ECT in the treatment of MDD. They proposed that a “connectivity resetting” takes place in which ECT resets abnormal neural connections after the induction of a generalized motor seizure. It may also modify delta and theta waves in the brain and increase blood flow to the thalamus (the brain’s “oscillation pacemaker”) to restore functional connections in the brain [89]. ECT appears similar to clozapine in its proposed action of inducing “microseizures,” and the effects of magnetic seizure therapy (MST) in the treatment of depression seem to reinforce this theory [86]. MST appears to work similarly to ECT by inducing seizures, and for some patients has resulted in significant improvement, with 40–60% of patients having a reduction in symptoms and 15–30% achieving remission [86]. Takamiya et al. [90] found that ECT-induced changes in right frontotemporal and thalamocortical connectivity and changes in the nodes of the default mode network were associated with clinical improvement, which further supports the modulation of neuronal networks to be an underlying mechanism by which ECT acts as an antidepressant. In a study on delivering ECS to the microtubule-associated protein 6 (MAP6) knockout (KO) mouse model of depression, Jonckheere et al. [91] found an increase in the survival and integration of neurons born before ECS treatment, and that ECS treatment induced an overall increase in synaptogenesis. In a study of whether changes in synaptic plasticity produced by ECS in rat hippocampal formation are sustained after treatment, a single ECS treatment resulted in rapid changes in structural plasticity, whereas repeated ECS treatment was found to produce lasting changes (3 months post-ECS) in synaptic plasticity and in non-neuronal plasticity, including an increased total length of microvessels and mitochondrial numbers [92]. Mitochondria have been found to play a critical role in BDNF-mediated synaptic and vascular plasticity in the hippocampus following ECS administration [93]. Increased gray matter volumes in the right hippocampus/amygdala region, involved in emotional processing and memory, have been found in patients post-ECT, which further supports increased neuroplasticity as a plausible mechanism of the antidepressant effect of ECT [94].

Alterations in dopaminergic neurotransmission have also been implicated as part of the mechanism by which ECT exerts its effects in depressive disorders, as shown in a study (n = 8) examining the dopamine transporters involved in the regulation of extracellular dopamine concentrations via pre- and post-ECT positron emission tomography (PET) [95]. The authors found the striatal dopamine transporter-binding potential was reduced in all patients following ECT. Other proposed mechanisms by which ECT may exert its effect in MDD include: changes in neurotransmission involving serotonin, acetylcholine, and norepinephrine; gene expression; alteration of blood–brain barrier permeability; increasing levels of neurotrophic factors; correcting glutamate/GABA imbalances; and neuroendocrine and neuroimmune modulation [81,91,96].

### 2.7. Clozapine and Parkinson’s Disease (PD)

Considerable evidence supports clozapine’s effectiveness in PD, primarily in the treatment of psychosis [97,98,99,100,101]. In two double-blind, randomized controlled trials, each involving 60 participants, low-dose clozapine (<50 mg/day) was found to be effective for managing symptoms of psychosis in PD patients and was well tolerated, without significant motor deterioration and with significantly improved symptoms of tremor [99,100]. Clozapine also demonstrated superiority versus the placebo, improving Global Impression Scales (GIS) and the Positive and Negative Symptom Scale (PANSS) scores in a study by Wilby et al. [97], suggesting that clozapine should be considered a first-line treatment option in PD-related psychosis. In a recent review paper on the treatment of psychosis in PD and in dementia with Lewy bodies, clozapine and quetiapine were reported to have some efficacy in both conditions (although the evidence for clozapine was more robust) and were relatively safe [99]. A review by Fox [102] found that clozapine significantly improved symptoms of PD, such as tremor and dyskinesia, due to its relationship with several neuroreceptors, including the 5HT1 receptor agonist and 5HT2A/2C receptor, and muscarinic receptor antagonism. Thomas and Friedman [103] reported clozapine’s usefulness in the treatment of both psychosis and tremor in PD, stating that it provided benefit to 82% of patients. Despite favorable evidence supporting its use, clinically, the use of clozapine is limited by the risk of agranulocytosis and monitoring requirements [99,104]. More evidence is required to determine the mechanism of action of clozapine in PD. Current preliminary evidence supports a role for D1 receptor antagonism and 5HT2A receptor antagonism, particularly in the context of psychosis and L-3,4-dihydroxyphenylalanine (L-DOPA)-induced dyskinesias [105].

### 2.8. ECT and PD

PD is a progressive neurodegenerative disorder that may be nonresponsive to pharmacotherapy, and ECT may play a role through the improvement of dopaminergic transmission, in turn reducing motor symptoms [106,107]. In addition, many patients with PD experience neuropsychiatric symptoms and comorbid psychiatric illness. Evidence supporting the impact of ECT in the treatment of PD has been present since the late 1940s, but much of the information is in the form of case reports. Similar to clozapine, ECT has been shown to be effective in PD for treatment-resistant motor symptoms, such as tremor and cogwheel rigidity, and in treating comorbid depression [106,108,109]. Calderon-Fajardo et al. [110] discussed the role of ECT in patients with PD and comorbid major depression, psychosis, or combined psychosis and depression. They demonstrated that ECT has the ability to significantly reduce both neuropsychiatric and motor symptoms in patients with PD. In a retrospective study (n = 12) by Murayama et al. [111] in advanced PD patients, the authors found ECT to be effective in reducing both severe motor symptoms (measured by the change in mean Hoehn and Yahr staging scores) and psychiatric symptoms (measured by the change in Neuropsychiatric Inventory scores). In addition, the authors also noted that impulse control disorders, which were present in five patients, were in remission following ECT. Likewise, a systematic review and meta-analysis by Takamiya et al. [109] summarizing the efficacy of ECT for symptoms in patients with PD identified 14 studies (n = 129) and reported that ECT significantly improved motor symptoms, even in the subgroup without psychiatric symptoms; improved depression and psychosis; reduced the wearing-off phenomenon of L-DOPA; and did not worsen cognition in PD patients. The authors stated that ECT was effective for PD patients with pharmacotherapy-resistant motor and psychiatric symptoms, which may distinguish ECT from other treatment options. In a case study, ECT was effective in the treatment of catatonia in PD with psychosis and was found to also improve motor symptoms [112]. Volkaerts et al. [113] reported improvement in both neuropsychiatric and motor symptoms in a PD patient with a functioning deep brain stimulator treated with ECT.

In a systematic review of the use of ECT in the treatment of depression in PD patients, Borisovskaya et al. [114] identified 43 articles and found that depression improved in 93% of those patients, and where motor symptom severity was reported, 83% improved. It has been proposed that following the production of a seizure, ECT increases L-DOPA penetration across the blood–brain barrier, increases serotoninergic neurotransmission and activation of mesocorticolimbic pathways, increases responses to dopamine, and increases blood flow and metabolism in specific areas of the brain, such as the hippocampus [110]. In animal models of PD, ECS is believed to improve symptoms by increasing dopamine release and modulating dopamine receptors in the striatum, in particular D1 and D3 receptor binding [115,116]. ECT is believed to also exert its effects in PD by increasing neuroplasticity and upregulation of BDNF in various brain regions, with increased hippocampal neurogenesis and mossy fiber sprouting [117]. In the rat hippocampus, enhanced production of endocytosis proteins and membrane trafficking machinery, which transport functional proteins in the neuronal cells, has been identified after 10 days of ECS treatment, suggesting a mechanism by which ECS increases neuroplasticity and results in symptom improvement in PD [117].

## 3. Other Factors Relevant to the Effects of Clozapine and ECT on Neuropsychiatric Disorders

There is now a large body of research into the immune system indicating the presence of cerebral inflammation in each of the disorders described above [118,119,120,121,122,123,124,125]. This inflammation has been proposed to be related to the release of inflammatory cytokines from activated glia [119,123,126,127,128,129,130,131]. Cytokine release can be further involved in increased glutamate release and the alteration of tryptophan metabolism (resulting in the production of several glutamate-like and free radical-generating metabolites) [132]. Although the focus of this glia-mediated inflammation has been on the microglia (the resident immune cells of the brain), more recent evidence has also indicated the involvement of astrocytes and oligodendrocytes [125,126,130,133,134]

There are now numerous reports in the literature indicating that both clozapine [135,136,137,138] and ECT [133,139,140,141,142,143] can reduce the release of inflammatory cytokines from glia. It is interesting that both treatments have dual effects on inflammation, initially increasing inflammation but decreasing it after longer-term administration [85,96,135,144]. It should be noted here that the literature on the involvement of the immune system and cytokines in neuropsychiatric disorders is not without some conflicting findings, and van Beul et al. [145] concluded that targeted potentiation rather than suppression of inflammatory responses might be relevant to the treatment of certain subgroups of depressed patients.

Another severe neuropsychiatric disorder in which both clozapine and ECT have been used is catatonia. Catatonia can be associated with a wide range of neuropsychiatric disorders and drug-induced conditions. There are three basic types, namely a stuporous form (mutism, rigidity, immobility, negativism, posturing, and catalepsy), an excited form (excitement, aggression, impulsivity), and an extreme form known as malignant catatonia (associated with autonomic instability and fever) [146]. The first-line drugs for the treatment of catatonia are benzodiazepines, with ECT also established as an effective treatment [146,147]. Although not used extensively for this purpose, it has been reported that clozapine is unique among antipsychotics by improving rather than causing or worsening the symptoms of catatonia [146,148,149,150]. Clozapine has been used in treatment-resistant cases of catatonia, i.e., cases that did not respond to benzodiazepines or ECT. Withdrawal catatonia has also been reported with the discontinuation of benzodiazepines and clozapine [151,152,153]. Although the neurobiology of catatonia is complex [154], GABAgeric hypoactivity is thought to play an important role [146]. Both ECT and clozapine have been reported to increase GABAergic activity, with ECT increasing serum GABA levels and GABA_B_ receptor activity, and clozapine also increasing activity of GABA_B_ receptors, increasing vesicular GABA transport, causing epigenetic effects on GABA gene promoters, and increasing GABA levels in the hippocampus and ventral tegmental areas of the brain (review: see [146]).

## 4. Conclusions

There appears to be significant overlap and synergism in the effectiveness of clozapine and ECT in various treatment-resistant disorders, including schizophrenia, MDD, bipolar disorder, and PD, prompting the hypothesis that common or related mechanisms exist between these two seemingly different treatment modalities (see Table 1 for a brief summary). A review of the current literature supports that the idea that the effectiveness of clozapine may be due to similarities in the mechanism to ECT in the following areas: epileptiform activity and EEG changes, increased neuroplasticity and levels of BDNF, effects on the glutamate/GABA imbalance that occurs in a variety of psychiatric and neurologic disorders, and influence on neuron–glia interactions by a reduction in inflammation through effects on cytokine release from glia. Both treatments are multifaceted, making comparisons difficult in areas such as the release and receptor activity with regard to biogenic amines. Early studies indicate that both treatments have interesting effects on epigenetics and connectomics [32,45,89,90,155,156,157,158,159], and, in the future, direct comparisons of clozapine and ECT in these two areas of endeavor should provide further valuable information about their mechanisms of action.

## Figures and Tables

**Table 1:** Summary of use of clozapine and ECT in neuropsychiatric disorders.

Area of Interest	Comments
Schizophrenia	Clozapine is the gold standard for therapy of treatment-resistant schizophrenia despite causing a plethora of side effects. ECT is being used increasingly in schizophrenia as a promising augmentation strategy with clozapine in clozapine-resistant schizophrenia.
Bipolar Disorder	Although data are limited, current evidence suggests that clozapine is effective and relatively safe to use in treatment-resistant bipolar disorder. ECT is considered a second-line option in refractory mania and a first-line option in severe I disorder, psychotic depression, and suicide risk.
Major Depressive Disorder (MDD)	Clozapine is not normally used as a primary treatment for MDD, but a large study [19] found a reduced risk of hospital readmission in patients with MDD taking clozapine; clozapine was reported to be more effective than quetiapine in treating depressive symptoms in those patients. ECT is a very effective treatment for MDD and is considered safe and well tolerated.
Parkinson’s Disease (PD)	Clozapine is effective in treating psychosis in PD but has also been reported to improve symptoms of tremor and dyskinesias.ECT has been found to reduce motor symptoms and to be effective in treating comorbid depression.
Catatonia	Catatonia can be associated with a wide variety of neuropsychiatric disorders and some drug-induced conditions. Benzodiazepines are the first-line treatment, but ECT is also an established effective treatment. Clozapine is not used extensively but appears to be unique among antipsychotics in that it improves the symptoms of catatonia rather than causing or worsening them.
Mechanisms of action	Although the mechanisms of action of clozapine and ECT are complex and still not clearly understood, a review of the literature on these two treatments suggests that the following may be common underlying mechanisms and may explain why both treatments are effective in a wide range of neuropsychiatric disorders: elicit epileptiform activity and alter EEG activity, increase neuroplasticity and cause an elevation of levels of neurotrophic factors, alter imbalances between GABAergic and glutamatergic systems, and reduce inflammation by acting on glia and cytokines.
